# LC-MS/MS identifies elevated imidazole propionate and gut-derived metabolite alterations in peritoneal dialysis patients

**DOI:** 10.1016/j.csbj.2025.11.039

**Published:** 2025-11-17

**Authors:** Weerawan Manokasemsan, Narumol Jariyasopit, Kwanjeera Wanichthanarak, Patcha Poungsombat, Alongkorn Kurilung, Suphitcha Limjiasahapong, Kajol Thapa, Yongyut Sirivatanauksorn, Sukit Raksasuk, Thatsaphan Srithongkul, Chagriya Kitiyakara, Sakda Khoomrung

**Affiliations:** a Department of Biochemistry, Faculty of Medicine Siriraj Hospital, Mahidol University, Bangkok, Thailand; b Siriraj Center of Research Excellent in Metabolomics and Systems Biology (SiCORE-MSB), Faculty of Medicine Siriraj Hospital, Mahidol University, Bangkok, Thailand; c Siriraj Metabolomics and Phenomics Center, Faculty of Medicine Siriraj Hospital, Mahidol University, Bangkok, Thailand; d Thailand Metabolomics Association, Bangkok, Thailand; e Department of Surgery, Faculty of Medicine Siriraj Hospital, Mahidol University, Bangkok, Thailand; f Division of Nephrology, Department of Medicine, Faculty of Medicine Siriraj Hospital, Mahidol University, Thailand; g Department of Medicine, Ramathibodi Hospital, Mahidol University, Bangkok, Thailand; h Center of Excellence for Innovation in Chemistry (PERCH-CIC), Faculty of Science, Mahidol University, Bangkok, Thailand

**Keywords:** LC-MS/MS, Quantitative metabolomics, Kidney failure, Peritoneal dialysis, Gut microbiota-derived metabolites, Uremic toxins

## Abstract

We developed a robust LC–MS/MS method for the simultaneous quantification of 16 uremic toxins (UTs) and 14 bile acids (BAs) in plasma and fecal samples within a single method. The method demonstrated high sensitivity, broad metabolite coverage, and excellent accuracy, precision, and throughput. Using this platform, targeted metabolites were quantified in peritoneal dialysis (PD) patients (n = 31) and healthy controls (HC; n = 60). Of the 30 targeted metabolites included in the validation method, 20 were detected in fecal samples and 12 in plasma in this study. Fecal samples exhibited greater BA diversity, whereas UTs were evenly distributed across both matrices. Fecal profiles showed minimal differences between PD and HC, suggesting limited gut-level alteration. In contrast, plasma analysis revealed nine metabolites significantly elevated in PD, including indoxyl sulfate, phenyl sulfate, hippuric acid, and imidazole propionate (ImP), lithocholic acid, cinnamoylglycine, m-hydroxyhippuric acid, phenylacetylglutamine, and phenylacetylglycine. Notably, plasma ImP—an underexplored metabolite—was elevated independently of diabetes or cardiovascular disease, implicating impaired renal clearance as its primary driver. These results highlight the systemic impact of gut-derived metabolites in kidney failure and position targeted UT–BA profiling as a powerful complementary tool for clinical metabolomics in chronic kidney disease and PD.

## Graphical Abstract

**Figure:**
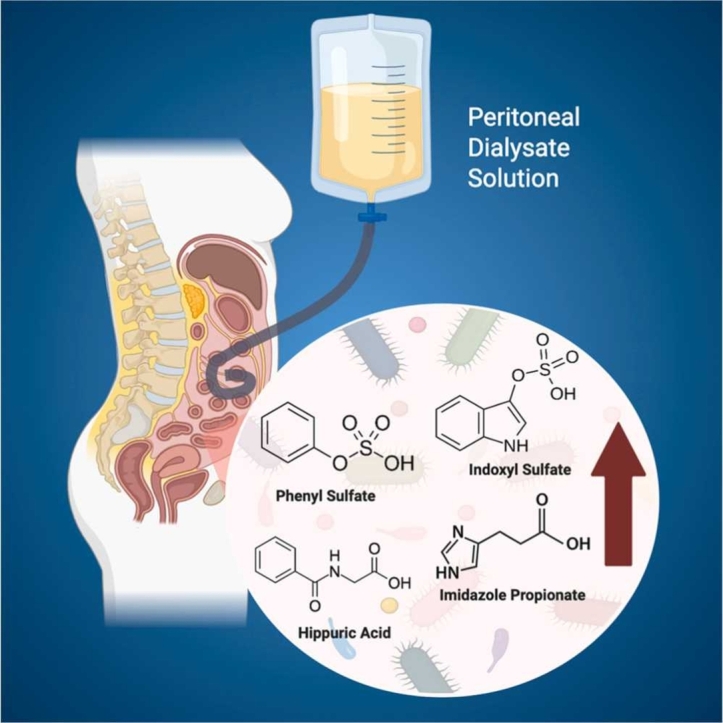

## 1 Introduction

Chronic kidney disease (CKD) is a major global health challenge, with many patients progressing to kidney failure (KF)—a condition associated with high morbidity and mortality [1]. The prevalence of KF requiring dialysis or transplantation has risen by over by 40 % in the past two decades [1]. Compared with hemodialysis (HD), peritoneal dialysis (PD) offers continuous uremic toxins removal, technical simplicity, home-based care, and cost-effectiveness, making it especially suitable for resource-limited settings [2]. In several countries, such as Thailand, PD has become the mainstay of kidney replacement therapy [3]. Despite advances in dialysis, 5-year survival remains low (42–52 %) [4], largely due to the incomplete clearance of uremic toxins (UTs). PD patients commonly accumulate *p*-cresol sulfate (*p*-CS), indoxyl sulfate (IS) [5], [6], and indole-3 acetic acid [7], which contribute to cardiovascular mortality and metabolic complications [8]. In CKD, elevated intraluminal urea, reduced pH, and impaired gut barrier function disrupt microbial balance and metabolism, leading to accumulation of UTs due to reduced renal clearance [9], [10], [11]. These gut microbiota-derived metabolites (GMMs) have emerged as key contributors to CKD pathophysiology, driving interest in therapies aimed at reducing their systemic burden [12], [13], [14]. However, the specific roles of GMMs—particularly in PD—remain largely unexplored. Phenyl sulfate (PS) promotes albuminuria in diabetic kidney disease (DKD) [15], and accumulates in HD patients [12], [13], [14], while imidazole propionate (ImP), a microbial product of histidine metabolism, has been linked to metabolic and cardiovascular disorders, including heart failure and increased mortality risk in non-CKD populations [16], [17], [18]. Given shared mechanisms such inflammation, oxidative stress, and metabolic dysregulation [19], [20], [21], [22], these metabolites may contribute to systematic complications in PD patients.

Beyond UTs, bile acids (BAs) are also central to host-microbe signaling. Gut microbes convert primary BAs (PBAs) into secondary BAs (SBAs) [23]. In CKD, dysbiosis not only increases UT production but also impairs the production of SBAs, reducing protective species that maintain barrier integrity and modulate inflammation [24]. Yet, their roles in KF remain underexplored.

Mass spectrometry (MS) is the preferred platform for GMM analysis [25], [26]. However, because hydrophobic BAs and hydrophilic UTs require distinct chromatographic conditions, most studies quantified them separately [12], [27], [28], [29], [30], [31]. BA analysis typically uses reversed-phase columns [32], [33], [34], where UTs are analyzed by hydrophilic interaction liquid column (HILIC) [28], complicating simultaneous detection. To overcome this, we developed and validated a single LC-MS/MS method capable of quantifying 14 BAs (four PBAs and 10 SBAs), and 16 UTs, in plasma and fecal samples from Thai PD patients, enabling integrated assessment of GMMs in KF.

## 2 Experimental procedures

### 2.1 Subjects and clinical samples

Thirty-one KF patients undergoing continuous ambulatory peritoneal dialysis using exclusively glucose-based peritoneal fluid solutions were recruited from the Peritoneal Dialysis clinic, Banphaeo General Hospital, Thailand. This study was approved by the Human Research Ethics Committee, Faculty of Medicine Ramathibodi Hospital, Mahidol University (COA. MURA2014/369). Clinical information, including diabetes and past cardiovascular history, were obtained from patient interviews and medical records. Healthy controls (HC, n = 60), were collected at King Chulalongkorn Memorial Hospital with approval from the Institutional Review Board of the Faculty of Medicine, Chulalongkorn University, Bangkok, Thailand (No. 057/62). All volunteers had normal kidney function, blood pressure, and no chronic illness medication uses. Written informed consent was obtained, and the studies were conducted according to the principles of the Declaration of Helsinki.

Blood samples were collected after an 8-hour fast for biochemical tests. Fecal and plasma samples were processed for metabolomics as previously described [35], immediately frozen at −80 °C, pooled (feces [100 mg from each individual] and plasma [100 µL from each individuals] separately) for method development and quality control (QC).

### 2.2 Chemicals and standards

Targeted analytes were verified with authentic standards (Table S1). Internal standards (IntStds) including *p*-cresol sulfate potassium salt-D_7_ (*p*-CS-d_7_,98 %) and glycodeoxycholic acid (GDCA-d_6_; 2,2,4,4,11,11-D_6_, 98 %) were used for quantitative analysis.

### 2.3 Extraction of GMMs from fecal and plasma samples

UTs and BAs in fecal and plasma samples were extracted using a unified protocol and adopted from a previous study [36]. A 250 mg fecal sample was extracted with 2 mL acetonitrile:methanol (ACN:MeOH, 75:25 v/v) containing 11.1 µM GDCA-d6. The mixture was vortexed for 20 s and centrifuged at 2500 ×g for 15 min at 4 °C. Supernatants were diluted (1:1 and 1:9) with ACN:MeOH (75:25 v/v) containing 20 µM *p*-CS-d_7_. A 100 µL of plasma sample was extracted with 900 µL of ACN:MeOH (75:25 v/v) containing 11.1 µM *p*-CS-d_7_ and 1.1 µM GDCA-d_6_. The mixture was vortexed for 20 s and centrifuged at 14,800 ×g at 4 °C for 15 min. After the centrifugation, 950 µL of the supernatant was transferred into an LC vial. An additional two dilutions were prepared: 1) 1:1 dilution where 100 µL of the supernatant was added with 100 µL of ACN:MeOH (75:25 v/v), and 2) 1:49 dilution where 30 µL the supernatant was diluted with 1470 µL of ACN:MeOH (75:25 v/v). An overview of the sample preparation and analysis protocol is given in Figure S1.

### 2.4 LC-MS/MS analysis

The analysis was performed on a Waters Xevo TQ-S UPLC–MS/MS system (Waters Corporation, Milford, MA, USA) using an ACQUITY BEH HILIC column (2.1 mm × 100 mm, 1.7 μm). Mobile phase A was ACN:H_2_O (95:5 v/v) with 10 mM ammonium formate (NH_4_HCO_2_) and 0.125 % of formic acid (CH₂O₂; pH 3); mobile phase B was ACN:H_2_O (50:50 v/v) with the same buffer. The gradient program started at 99 % A from 0 to 1 min, decreased to 65 % A at 10 min, and returned to 99 % A for re-equilibration from 11 to 15 min. The flow rate was 0.4 mL/min, the injection volume was 5 μL, and the column temperature was maintained at 45 °C [36]. Mass spectrometric detection was performed in ESI⁺ and ESI⁻ modes (capillary 2.0 kV, cone 40 V, source 150 °C, desolvation 500 °C, gas 1000 L/h, cone gas 150 L/h, nebulizer 6.5 bar).

### 2.5 Method validation

#### 2.5.1 Sensitivity and precision

Limit of detection (LOD) and limit of quantification (LOQ) were determined following a published protocol [37]. A twelve-points matrix-matched calibration curve (0.005 µM to 20 µM) containing 34 mixed standards and two IntStds were analyzed in triplicates, yielding correlation coefficients (R² > 0.99). Precision and reproducibility were evaluated using 10 µM mixed standards of UTs and BAs analyzed in 10 replicates within a day (intra-day) and over three consecutive days (inter-day) [35]. The mean and % relative standard deviation (RSD) of retention time and peak area were calculated, with an acceptance criterion of ≤ 20 %RSD [38].

#### 2.5.2 Accuracy and matrix effect

Recoveries were assessed by comparing pre- and post-spiked samples (10 µM, *n* = 3) using matrix-matched calibration curves [39].

$$\%\mathrm{Recovery}=\;\frac{\mathrm{Pre}-\mathrm{spiked}\;\mathrm{concentration}\;\times \;100\%}{\mathrm{Post}-\mathrm{spiked}\;\mathrm{concentration}}$$

Matrix effects were evaluated by comparing calibration slopes (R^2^ > 0.99) obtained in neat solvent (in normal calibration) and matrix extracts,percent matrix effect (%ME) was calculated as follows [35].

$$\%\mathrm{ME}=\;\frac{(\mathrm{slope}\;of\;m\mathrm{atrix}-\mathrm{matched}\;\mathrm{calibration})\;\times \;100\%}{\left(\mathrm{slope}\;\mathrm{of}\;\mathrm{normal}\;\mathrm{calibration}\right)}$$

### 2.6 Quantitative analysis of UTs and BAs in fecal and plasma samples

Quantitation was performed using matrix-matched calibration curves [39]. Mixed standards were serially diluted in pooled matrix extracts (0.005 – 20 µM, 10 points) and analyzed in triplicate. The IntStds (*p*-CS-d_7_ and GDCA-d_6_) were added to achieve final concentrations of 10 µM and 1 µM for the ESI⁻ and ESI⁺ modes, respectively (20 µL of 500 µM *p*-CS-d_7_ and 2 µL of 500 µM GDCA-d_6_). Calibration curves showed excellent linearity (R² > 0.99).

For quality assurance and QC, pooled plasma and fecal samples were processed alongside the samples and injected across all analytical batches. Samples were analyzed in random order to minimize batch effects.

### 2.7 Data processing and analysis

LC-MS/MS data were processed using Targetlynx informatics in Masslynx software (version 4.1 SCN950; Waters Corporation, Milford, MA, USA). Metabolite identification was confirmed by matching retention time, precursor, and fragmented ions with authentic standards following the Metabolomics Standards Initiative Level 1 criteria [40].

Metabolites detected in fewer than 80 % of samples within each group were excluded from the analysis. For metabolites that met this criterion, missing values due to undetected or unquantifiable peaks were imputed with the minimum value of the corresponding group. Samples showing recoveries below 80 % or above 120 %, based on back-calculated IntStd concentrations, were considered to be affected by matrix effects. These samples were reanalyzed, and similar results were obtained. Therefore, their values were imputed using the group median [41]. The imputed data were used in further analysis (Table S2). The univariate analysis was using GraphPad Prism software (version 8.4.2). Statistical comparisons across groups were performed using linear models, accounting for age, sex, and groups. Principal component analysis (PCA) was performed using SIMCA software (version 18.0.1.507). Spearman correlation analysis adjusted by Benjamini & Hochberg was conducted by Metabox 2.0 [41].

## 3 Results

### 3.1 LC-MS/MS setup and validation

**Fig. 1:**
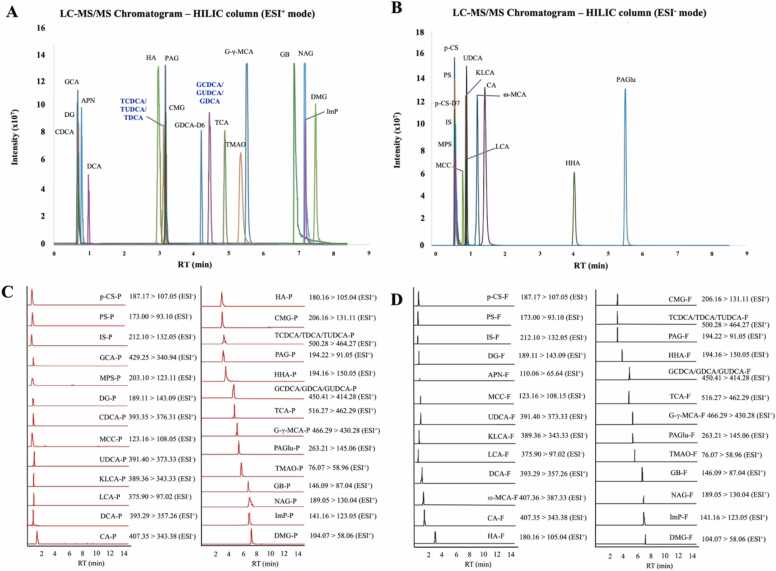
Overview of LC-MS/MS analysis of target compounds using a HILIC normal phase column. All the single authentic standards were individually analyzed in A) positive ESI (ESI^+^) mode and B) negative ESI (ESI^-^) mode. MRM transitions of all targets detected are shown for C) plasma and D) fecal samples. The label after compound abbreviation represents the sample types: “-P” represents analytes in plasma samples and “-F” represents analytes in fecal samples. Abbreviations: *p*-CS – *p*-Cresol sulfate; PS – Phenyl sulfate; IS – Indoxyl sulfate; GCA – Glycocholic acid; MPS – 2-Methoxyphenylsulfate; DG – Diethyl glutarate; APN – 2-Aminophenol; CDCA – Chenodeoxycholic acid; MCC – 4-Methylcatechol; UDCA – Ursodeoxycholic acid; KLCA – 12-ketolithocholic acid; LCA – Lithocholic acid; DCA – Deoxycholic acid; ω-MCA – ω-Muricholic acid; CA – Cholic acid; HA – Hippuric acid; CMG – Cinnamoylglycine; TCDCA – Taurochenodeoxycholic acid; TDCA – Taurodeoxycholic acid; TUDCA – Tauroursodeoxycholic acid; PAG – Phenylacetylglycine; HHA – m-Hydroxyhippuric acid; GCDCA – Glycochenodeoxycholic acid; GDCA – Glycodeoxycholic acid; GUDCA – Glycoursodeoxycholic acid; TCA – Taurocholic acid; G-γ-MCA – Glyco-γ-muricholic acid; PAGlu – Phenylacetylglutamine; TMAO – Trimethylamine N-oxide; GB – 4-Guanidinobutyric acid; NAG – N-Acetylglutamine; ImP – Imidazolepropionic acid; DMG – Dimethylglycine; RT – Retention time.

Cone voltage and collision energy were optimized for each compound using [M+H]^+^ or [M-H]^-^ parent ions. As trimethylamine N-oxide (TMAO), *p*-CS-d_7_, and GDCA-d_6_, were undetectable in auto-infusion mode, parent and fragment ions were predicted using Competitive Fragmentation Modeling for Metabolite Identification (https://cfmid.wishartlab.com/predict) and verified by manual infusion of authentic standards (Table S3). Reversed-phase chromatography (HSS T3 C18 column) detected 12 of 33 standards in ESI^+^ mode and 19 in ESI^-^ (Figures S2A, S2B). 12-Ketolithocholic acid and diethyl glutarate (DG) were undetectable in both mode, and several analytes eluted within 0.5–0.6 min, complicating identification and quantification. In contrast, the HILIC method detected all 33 targets (21 in ESI^+^ and 12 in ESI^-^; Figures 1A, 1B, Table S3). However, co-elution of (i) taurochenodeoxycholic acid (TCDCA), taurodeoxycholic acid (TDCA), and tauroursodeoxycholic acid (TUDCA); and (ii) glycochenodeoxycholic acid (GCDCA), glycodeoxycholic acid (GDCA), and glycoursodeoxycholic acid (GUDCA), limited the separation efficiency. The HILIC method provided optimal separation within 15 min and higher sensitivity compared to the HSS T3 C18 column and was therefore selected for GMM quantification, detecting 27 analytes in plasma (Fig. 1C) and 26 analytes in fecal samples (Fig. 1D).

Method validation results are summarized in Table S4. The LOD and LOQ ranged from 0.240 nM to 2.259 µM, and from 0.780 nM to 7.529 µM, respectively. Intraday %RSD of peak area ranged from 1.14 % to 16.39 %, and interday %RSD from 0.76 % to 18.60 %. Retention time %RSD of both intraday and interday experiment remained below 7.81 %, meeting Association of Official Analytical Collaboration (AOAC) precision criteria (%RSD < 21 %) [38], [42]. The quantification of matrix effects of all analytes ranged from 0.4 % to 141.90 %, observed in both feces and plasma. Percent recoveries of the targeted compounds in fecal matrix varied from 78.54 % to 117.18 %, with chenodeoxycholic acid (CDCA) slightly exceeding the FDA-recommended range (80–120 %) [43]. In plasma, percent recoveries ranged from 79.42 % to 115.68 %, with all values but DG falling within the FDA-accepted range (80–120 %) [43].

During sample analysis, three analytes—glycocholic acid, CDCA, and GB—were found to accumulate on the column and were thus excluded. Additionally, conjugated bile acids (GCDCA, GDCA, GUDCA, TCDCA, TDCA, and TUDCA) were quantified into taurine- and glycine-conjugation due to co-elution (Fig. 1A), reducing final targets to 26 analytes (10 BAs, 16 UTs).

### 3.2 Cohort characteristics

Sixty HC participants and thirty-one PD patients were enrolled, with clinical data available for 57 HC and 28 PD patients (Table 1 and full detail in Table S5). The PD vintage ranged from 3 months to 8 years. Median ages were 22 years (range: 19–57) for HC and 58 years (range: 29–82) for PD. The HC group included 32 (56 %) females and 25 (44 %) males, while the PD group comprised seven (25 %) females and 21 (75 %) males. The median body mass index of PD patients was 22.5 ± 2.9 kg/m².

**Table 1:** Summary of cohort characteristics.

**Cohort Characteristics**	**HC (n = 57)**	**PD (n = 28)**	**Statistical*****p***-**value**
**Age (years)**	22 (19−57)	58 (29−82)	< 0.0001
**Sex**			
Female	32 (56.1 %)	7 (25.0 %)	0.00677
Male	25 (43.9 %)	21 (75.0 %)	
**BMI (kg/m**^**2**^**)**	-	22.5 ± 2.9	-
**Blood chemistry test**			
Fasting Plasma Glucose (mg/dL)*	82.0 ± 3.0	-	-
Cholesterol (mg/dL)*	193.0 ± 17.0	192.0 ± 29.0	ns
LDL (mg/dL)*	121.0 ± 14.0	98.0 ± 20.0	ns
HDL (mg/dL)*	56.0 ± 7.0	44.0 ± 11.0	ns
Triglyceride (mg/dL)*	65.0 ± 15.0	152.0 ± 63.0	< 0.0001
Creatinine (mg/dL)*	-	8.5 ± 4.0	-
Ferritin (ng/mL)*	-	578.2 ± 347.9	-
BUN (mg/dL)*	-	47.2 ± 14.7	-
Albumin (g/L)*	-	35.1 ± 4.0	-
PTH (pg/mL)*	-	146.5 ± 50.7	-
K^+^ (mmol/L)*	-	3.9 ± 0.5	-
**DM**	-	10 (35.7 %)	-
**DM-CVD**	-	16 (57.1 %)	-

Full details are provided in Table S5.

The statistical analysis of sex distribution was performed using the chi-square test.

* These data are reported in median ± median absolute deviation (MAD).

Blood chemistry data are reported as median ± median absolute deviation. Fasting plasma glucose, measured only in HC, was 82.0 ± 3.0 mg/dL. HC individuals had higher cholesterol, low-density lipoprotein, and high-density lipoprotein levels than PD patients, though differences were not statistically significant. However, PD patients had significantly higher triglyceride levels (152.0 ± 63.0 mg/dL) compared to HC individuals (65.0 ± 15.0 mg/dL, p < 0.0001). Additional clinical parameters available only for PD patients included creatinine, ferritin, blood urea nitrogen (BUN), albumin, parathyroid hormone (PTH), and potassium (K^+^) levels. In PD patients, the median creatinine level was 8.5 ± 4.1 mg/dL, ferritin was 578.2 ± 347.9 ng/mL, BUN was 47.2 ± 14.7 mg/dL, albumin was 35.1 ± 4.0 g/L, PTH was 146.5 ± 50.7 pg/mL, and K^+^ was 3.9 ± 0.5 mmol/L. Among the PD patients, 10 (35.7 %) were diagnosed with diabetes mellitus (DM) only, and 16 (57.1 %) had either DM, cardiovascular disease (CVD), or both.

### 3.3 Distinct plasma GMM signatures in PD vs. HC

**Fig. 2:**
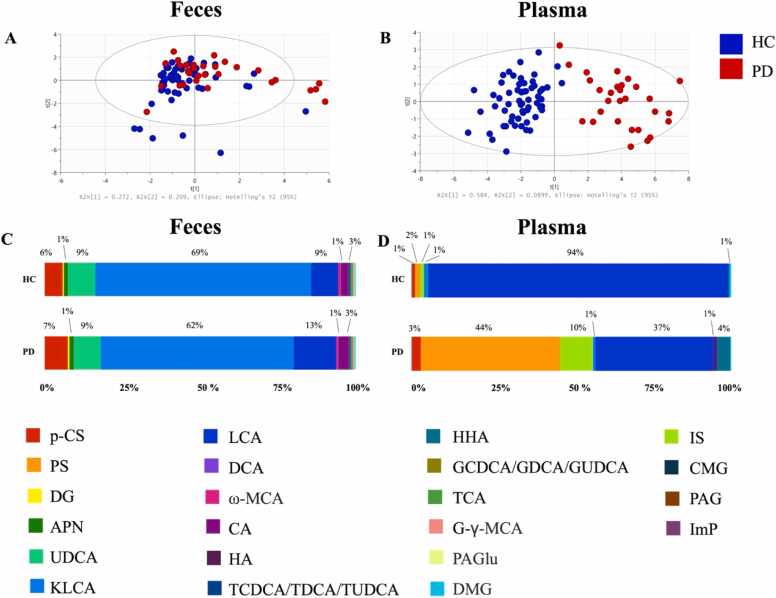
Observation of host-microbe co-metabolites in feces and plasma samples of HC compared to PD patients. PCA analysis is shown for the distribution of (A) fecal and (B) plasma host-microbe co-metabolites from HC (in blue) and PD patients (in red). The horizontal bar graph presents the proportion of (C) fecal and (D) plasma host-microbe co-metabolites in HC (upper bar) and PD patients (lower bar). Each color represents each analyte as mentioned in the legend. Abbreviations: *p*-CS – *p*-Cresol sulfate; PS – Phenyl sulfate; IS – Indoxyl sulfate; DG – Diethyl glutarate; APN – 2-Aminophenol; UDCA – Ursodeoxycholic acid; KLCA – 12-ketolithocholic acid; LCA – Lithocholic acid; DCA – Deoxycholic acid; ω-MCA – ω-Muricholic acid; CA – Cholic acid; HA – Hippuric acid; CMG – Cinnamoylglycine; TCDCA – Taurochenodeoxycholic acid; TDCA – Taurodeoxycholic acid; TUDCA – Tauroursodeoxycholic acid; PAG – Phenylacetylglycine; HHA – m-Hydroxyhippuric acid; GCDCA – Glycochenodeoxycholic acid; GDCA – Glycodeoxycholic acid; GUDCA – Glycoursodeoxycholic acid; TCA – Taurocholic acid; G-γ-MCA – Glyco-γ-muricholic acid; PAGlu – Phenylacetylglutamine; ImP – Imidazolepropionic acid; DMG – Dimethylglycine.

**Fig. 3:**
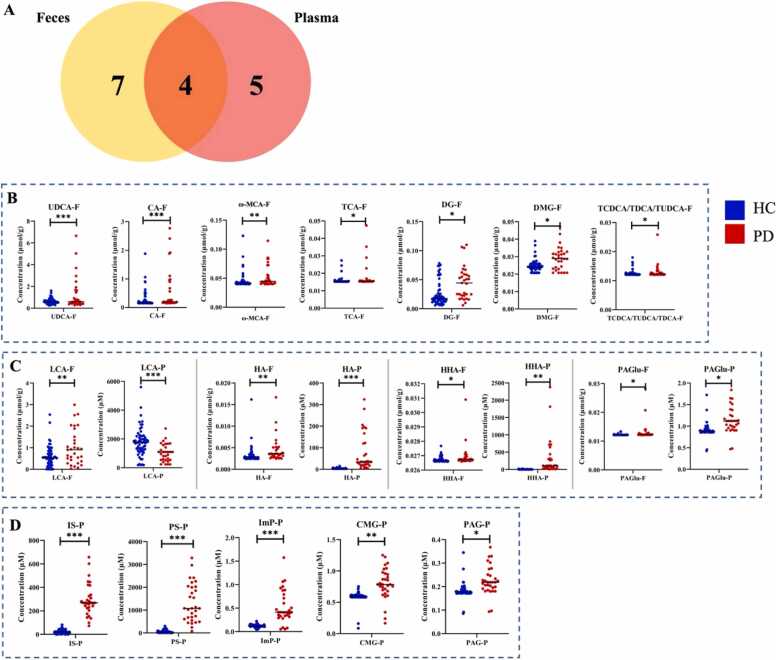
(A) Venn diagram presenting the number of significant metabolites in (B) feces samples only, (C) both fecal and plasma matrices, and (D) plasma samples only. Dot plots indicate the range of absolute concentrations of significant host-microbe co-metabolites in fecal (μmol/g) and plasma (μM) samples of 60 HC individuals (in blue) and 31 PD patients (in red). The statistical analysis was performed using a linear model with age, sex, and group parameters. The levels of significance are: *p < 0.05; **p < 0.01; ***p < 0.001; and ****p < 0.0001. Abbreviations: PS – Phenyl sulfate; IS – Indoxyl sulfate; DG – Diethyl glutarate; UDCA – Ursodeoxycholic acid; LCA – Lithocholic acid; ω-MCA – ω-Muricholic acid; CA – Cholic acid; HA – Hippuric acid; CMG – Cinnamoylglycine; TCDCA – Taurochenodesoxycholic acid; TDCA – Taurodeoxycholic acid; TUDCA – Tauroursodeoxycholic acid; HHA – m-Hydroxyhippuric acid; TCA – Taurocholic acid; PAGlu – Phenylacetylglutamine; ImP – Imidazolepropionic acid; DMG – Dimethylglycine; PAG – Phenylacetylglycine.

Because the extraction workflow includes an organic solvent–based protein precipitation step, the LC–MS/MS analysis quantifies total metabolite concentrations (free and protein-bound). Among the 30 targeted metabolites, 20 were quantified in fecal samples and 12 in plasma samples (Table S6). PCA shows similar fecal metabolite profiles between PD and HC group (Fig. 2A), but clear separation in plasma (Fig. 2B). Relative compositions further highlighted these differences (Fig. 2C and D): in HC plasma, lithocholic acid (LCA) represented 94 % of total metabolites, decreasing to 37 % in PD, while PS increased from 2 % in HC to 44 % in PD, and IS increased approximately ten-fold. A Venn diagram (Fig. 3A) illustrates the distribution of significant metabolites across fecal and plasma samples: seven were unique to feces, four shared, five plasma-specific. Fecal-only metabolites included ursodeoxycholic acid, cholic acid, ω-muricholic acid, taurocholic acid, DG, dimethylglycine (DMG), and TCDCA/TDCA/TUDCA (Fig. 3B). Shared metabolites were LCA, hippuric acid (HA), m-hydroxyhippuric acid (HHA), and phenylacetylglutamine (PAGlu) (Fig. 3C). Median concentrations of HA, HHA, and PAGlu were significantly higher in both fecal and plasma samples from PD patients compared to HC. While fecal LCA levels were higher in PD, plasma LCA levels were lower compared to HC (Fig. 3C). Plasma-specific metabolites included IS, PS, ImP, cinnamoylglycine (CMG), and phenylacetylglycine (PAG) (Fig. 3D).

### 3.4 Elevated levels of Plasma PS and ImP in KF

Using a linear model adjusted for group, sex, and age, nine metabolites were significantly (p-value < 0.01) altered between PD and HC (Table S6). The most significant were IS (p-value: 8.65E-12), PS (p-value: 6.06E-10), HA (p-value: 1.48E-07) and ImP (p-value: 4.32E-07), all elevated in PD. IS and HA are well-established UTs in KF [6], [44], [45], while PS and ImP have been less explored. Because our HC and PD cohorts were not age-matched, we performed an additional analysis restricted to participants aged > 40 years using the Mann–Whitney *U* test corrected by Holm-Sidak correction (Table S7). The differences in plasma PS and ImP levels between HC (n = 6) and PD (n = 26) remained significant (p < 0.0001 for PS and p = 0.002 for ImP).

**Fig. 4:**
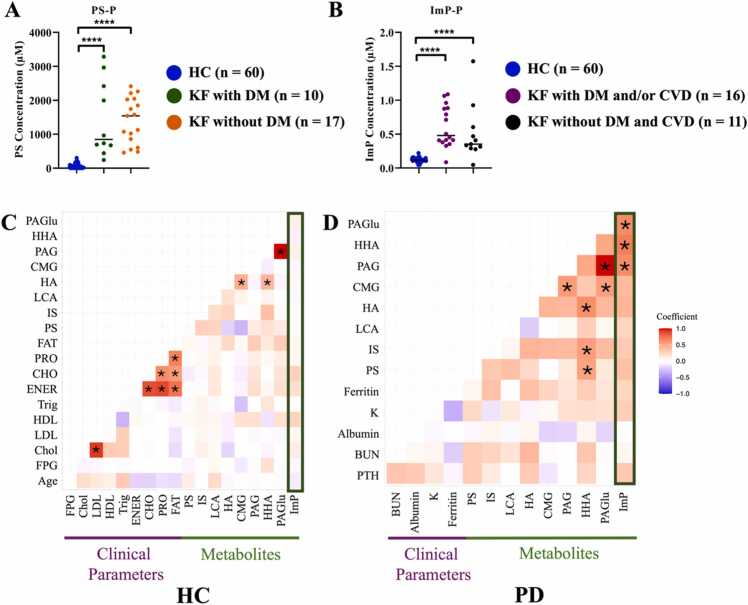
Dot plots compare PS content (A) in KF patients with DM (KF with DM) and without DM (KF without DM) and ImP content (B) in KF patients with DM, cardiovascular disease (CVD) or both (KF with DM and/or CVD) and and KF patients with neither DM nor CVD (KF without DM and CVD). The linear model adjusted with age, gender, and group were performed in comparison of HC and KF with different comorbidities, and “****” indicates *p* < 0.0001. Spearman correlations of significant plasma host-microbe co-metabolites and clinical parameters are shown for (C) HC individuals and (D) PD patients. The significance level was analyzed using Benjamini & Hochberg adjustment, and “*” indicates *p* < 0.05. Red indicates positive correlation and blue represents negative correlation. Additional abbreviations: FPG – Fasting plasma glucose; Chol – Blood cholesterol; LDL – Low-density lipoprotein; HDL – High-density lipoprotein; Trig – Triglyceride; ENER – Energy; CHO – Carbohydrate; PRO – Protein; FAT – Fat; PTH – Parathyroid hormone; BUN – Blood urea nitrogen; K – Potassium.

To determine whether PS and ImP elevations were KF-specific or related to comorbidities, subgroup analyses were performed. PS and ImP levels were comparable between patients with and without comorbidities (Fig. 4A–B). PS levels showed no significant difference between KF patients with (n = 10) and without DM (n = 17), although both groups had significantly higher PS levels compared with HC (n = 60; Fig. 4A). Similarly, ImP levels did not differ significantly between KF patients with DM, CVD, or both (n = 16), and those are neither DM nor CVD (n = 11), while all KF groups exhibited significantly higher ImP levels than HC (n = 60; Fig. 4B). No significant Spearman correlations (Fig. 4C–D) were found between PS and clinical parameters or other UTs, except for HHA in PD group (Fig. 4D), consistent with previous HD studies [12]. Based on these results, subsequent analyses focused on ImP. Spearman correlations (Fig. 4C–D) showed that in PD patients, ImP correlated positively with PAGlu, HHA, and PAG, while no associations were observed in HC participants.

To assess the clinical relevance, ImP was collated with ferritin, BUN, albumin, PTH, and K^+^ (Figure S3A-E). Ferritin, BUN, PTH, and K⁺ showed weak positive, non-significant associations, while albumin was inversely correlated with no significance, suggesting potential links between ImP and markers of kidney dysfunction and mineral metabolism.

## 4 Discussion

### 4.1 Methodological advances in LCMS/MS analysis

This study established a single LC-MS/MS method for simultaneous quantification of 14 BAs (4 PBAs and 10 SBAs) and 16 UTs in fecal and plasma samples. The approach broadened metabolite coverage, shortened analysis time, and enabled a unified assessment of metabolic alterations in PD patients. The HSS T3 C18 column exhibited limitations for hydrophilic compounds such as GB, APN, DMG, TMAO, NAG, and ImP, while the HILIC column achieved effective separation within 15 min and higher sensitivity. Although certain conjugated BAs (GCDCA, GDCA, GUDCA, TCDCA, TDCA, and TUDCA) remained unresolved, the HILIC method offered great efficiency and was selected for the cohort analysis. Compared with eleven previously published LC-MS/MS methods (Table S8), this method demonstrated superior sensitivity, metabolite coverage, and throughput. For example, LOQ for *p*-CS was 1.84 nM —approximately threefold lower than previously reported values [27]. This enhanced sensitivity is essential for detecting low-abundance metabolites, improving signal-to-noise ratios, and increasing both measurement precision and reliability of clinical metabolomics interpretation. Beyond its high sensitivity, the method is straightforward and relatively high throughput: up to 240 samples can be processed within a few hours, requiring an average of only five min per sample.

This marks a substantial improvement over earlier protocols, which typically required between one hour and an entire day [27], [46], [47], and in some cases involved additional derivatization steps [48]. Importantly, when applied to clinical samples, the method is expected to deliver reliable quantitative results even in patients with severe hypoalbuminemia (serum albumin 14.1–54.6 g/L). Such conditions can alter plasma matrix composition, potentially affecting metabolite ionization efficiency and compromising quantitative accuracy, as previously observed across various sample types [35], [49], [50]. To mitigate these effects, matrix-matched calibration curves were employed to compensate for inter-individual differences in protein content and matrix complexity, thereby ensuring consistent quantification across the PD cohort.

### 4.2 Fecal and plasma metabolic profiles in PD and HC

To evaluate gut–kidney interactions, metabolites were analyzed in both fecal and plasma samples. Because GMMs mostly originate in the intestine before entering circulation, this dual analysis captured both local gut activity and systematic effects. Fecal samples contained a broader range of BAs (10 types), whereas plasma contained only two (Table S6). In contrast, a similar number of UTs (10) were detected in both matrices (Table S6). Plasma GMM profiles differed significantly between PD patients and HC, while fecal profiles were largely comparable (Fig. 2). These results suggest that kidney dysfunction mainly impacts systemic metabolism, leading to the accumulation of GMMs in plasma. As most uremic toxins are absorbed and renally excreted rather than eliminated via stool, plasma offers a clearer distinction between PD and HC and serves as a more practical matrix for biomarker discovery and clinical monitoring. Blood collection is routine in clinical settings and better suited for long-term monitoring and clinical trials. In contrast, fecal sample collection poses logistical challenges, as it requires patients to follow specific handling and transport procedures [35]. Nevertheless, for research focused on bile acid metabolism and its association with CKD, fecal samples remain essential for a comprehensive analysis.

### 4.3 Phenyl sulfate and imidazole propionate as emerging uremic toxins

We identified nine plasma metabolites that were significantly elevated in PD patients: IS, PS, HA, ImP, LCA, CMG, HHA, PAGlu, and PAG. Except for PS and ImP, these metabolites are well-documented in CKD and KF [15], [51], [52], [53], [54], [55]. To minimize potential bias from age differences between cohorts, age was included as a covariate in the linear model. Furthermore, subgroup analysis restricted to participants aged over 40 years confirmed that PS and ImP levels remained significantly higher in PD patients than in HC, suggesting that the observed differences were not confounded by age. PS is derived from dietary tyrosine by gut bacteria and has been linked to kidney injury and the progression of albuminuria in pre-dialysis DKD patients [15], [56]. Previous studies have documented elevated PS in HD patients [57], [58], but its presence and clinical relevance in PD patients have not yet been investigated. Although 35.7 % of our PD patients had DM, PS concentrations did not significantly differ between patients with and without DM, indicating its elevation is likely associated with KF independently of diabetes.

ImP, another gut-derived metabolite, has been shown to disrupt insulin signaling through mTORC1 activation [16]. Prior studies have linked ImP to kidney dysfunction and metabolic issues in DKD [55], [59] as well as to the development of type 2 diabetes (T2DM) and atherosclerosis [60]. A recent study demonstrated that ImP promotes atherosclerosis in mice and correlates with disease severity in non-CKD cohorts, independent of lipid levels, through activation of systemic and local immune responses [61]. mdoel levels did not differ between PD patients with or without these comorbidities. Furthermore, ImP showed significant positive correlation with three other metabolites—PAGlu, HHA, and PAG—all of which are known markers of advanced kidney failure [12], [52], [62]. These results suggest that ImP accumulation occurs in PD patients as a function of kidney failure even in the absence of diabetes or diagnosed CVD.

### 4.4 Clinical associations of plasma imidazole propionate

Plasma ImP showed a significant inverse correlation with serum albumin, a well-known predictor of mortality and cardiovascular morbidity in dialysis patients. This finding suggests that elevated ImP may be associated with adverse clinical outcomes in PD [63], [64], [65], consistent with prior reports linking high ImP levels to heart failure and increased mortality in non-CKD populations [18]. ImP also correlated positively with PTH, a marker of secondary hyperparathyroidism commonly seen in advanced CKD and associated with mineral–bone homeostasis, cardiovascular risk, and mortality [66], [67]. These correlations underscore ImP’s potential clinical relevance as a potential biomarker of disease severity. Although preliminary, these findings indicate that plasma ImP is linked to adverse laboratory parameters. Larger, well-characterized studies incorporating dialysis clearance, residual kidney function, and dietary factors are needed to clarify the role of ImP as a potential UT in KF. Further work should also define determinants of ImP levels in PD patients and their relationships with systemic inflammation, protein-energy wasting, and cardiovascular risk.

## 5 Conclusion

This study established a high-sensitivity LC–MS/MS method for simultaneous quantification of multiple UTs and BAs in plasma and fecal samples. Nine plasma metabolites were significantly altered in PD patients, reflecting limited clearance of gut-derived solutes. Among these, ImP and PS emerged as novel and clinically relevant candidates. ImP, previously underexplored in KF, correlated with other UTs and adverse laboratory markers, suggesting poor dialysis clearance. Together, these findings underscore the systemic impact of GMMs in patients with KF and demonstrate the utility of this LC–MS/MS platform for clinical metabolomics in CKD and PD.

## 6 Study limitations and future directions

### 6.1 Study limitations

***Analytical limitations:*** Although the developed LC–MS/MS method demonstrated high sensitivity and throughput, it could not fully resolve several structurally similar conjugated bile acids—*GCDCA*, *GDCA*, *GUDCA*, *TCDCA*, *TDCA*, and *TUDCA*—which exhibited partial co-elution under optimized HILIC conditions. Complete separation of these bile acids would require a complementary reverse-phase method with tailored chromatographic gradients and ionization settings.

***Sample size and demographics:*** The relatively small PD cohort and demographic differences (age, sex) between PD patients and HC may reduce statistical power and generalizability.

***Clinical data gaps:*** Residual kidney function and dialysis adequacy were not assessed, although both are key determinants of UT clearance and may influence metabolite concentrations.

***Cohort specificity:*** All participants were Thai PD patients on glucose-based dialysate under the “PD-first” policy, typically associated with low residual kidney function. Therefore, the results may not fully represent patients treated with alternative PD regimens (e.g., icodextrin or amino acid–based dialysates) or those from other ethnic and dietary backgrounds.

***Microbiome profiling:*** Gut microbial composition was not analyzed, limiting insight into microbial pathways responsible for producing metabolites such as ImP and PS.

***Study design*****:** The cross-sectional design precludes causal inference and limits assessment of ImP and PS as predictive biomarkers.

### 6.2 Future directions

Building on this work, future studies should combine multi-omics approaches—including metagenomics, metatranscriptomics, and targeted metabolomics—to elucidate microbial and host pathways driving ImP and PS production and accumulation. Incorporating larger, multi-center, and longitudinal cohorts will be essential for validating ImP and PS as novel UTs and assessing their prognostic and therapeutic relevance in CKD and dialysis. From an analytical standpoint, expanding the current LC–MS/MS platform to include orthogonal separation modes (e.g., reversed-phase or ion mobility–MS [36]) will improve resolution of co-eluting bile acids and enable deeper metabolic coverage. Integrating this quantitative platform with intervention studies—such as dietary modulation, prebiotics/probiotics, or dialysis regimen optimization—could reveal actionable strategies to mitigate the systemic accumulation of gut-derived toxins and improve clinical outcomes in KF.

## CRediT authorship contribution statement

**Sukit Raksasuk:** Writing – review & editing, Supervision, Investigation, Funding acquisition, Conceptualization. **Kajol Thapa:** Writing – review & editing, Methodology, Investigation, Formal analysis, Data curation. **Yongyut Sirivatanauksorn:** Writing – review & editing, Supervision, Resources, Funding acquisition, Conceptualization. **Alongkorn Kurilung:** Writing – review & editing, Methodology, Investigation, Data curation. **Suphitcha Limjiasahapong:** Writing – review & editing, Methodology, Investigation, Formal analysis. **Kwanjeera Wanichthanarak:** Writing – review & editing, Writing – original draft, Supervision, Methodology, Investigation, Formal analysis. **Patcha Poungsombat:** Writing – review & editing, Visualization, Methodology, Investigation, Formal analysis. **Sakda Khoomrung:** Writing – review & editing, Writing – original draft, Supervision, Resources, Project administration, Methodology, Investigation, Funding acquisition, Formal analysis, Conceptualization. **Weerawan Manokasemsan:** Writing – review & editing, Writing – original draft, Validation, Project administration, Methodology, Investigation, Formal analysis, Data curation, Conceptualization. **Narumol Jariyasopit:** Writing – review & editing, Writing – original draft, Supervision, Methodology, Investigation. **Thatsaphan Srithongkul:** Writing – review & editing, Supervision, Investigation, Formal analysis. **Chagriya Kitiyakara:** Writing – review & editing, Writing – original draft, Supervision, Project administration, Investigation, Funding acquisition, Formal analysis, Conceptualization.

## Ethics approval and consent to participate

All the experiment and sample collection protocols were approved by Institutional Review Board of the Faculty of Medicine, Chulalongkorn University, Bangkok, Thailand (IRB No. 057/62) and the Human Research Ethics Committee, Faculty of Medicine Ramathibodi Hospital, Mahidol University (COA. MURA2014/369). This study was conducted in compliance with the Helsinki Declaration. Fecal and blood plasma samples were collected from all volunteers and patients with informed consent from all subjects.

## Funding

This research has received funding support from the NSRF via the Program Management Unit for Human Resources & Institutional Development, Research and Innovation [grant number B36G660007]. This research project has been supported by Mahidol University (Fundamental Fund: fiscal year 2025 by National Science Research and Innovation Fund (NSRF), to S.K.), Bangkok, Thailand. This study was also supported by a Siriraj Graduate Scholarship awarded to WM. The research project was partially supported by Scholarships for Ph.D. Student from Mahidol University (to S.K., W.M., K.T.). The authors acknowledge the Center of Excellence for Innovation in Chemistry (PERCH-CIC) and the Ministry of Higher Education, Science, Research, and Innovation, Thailand. This project is partially supported by the Research Excellence Developement (RED) program at the Faculty of Medicine Siriraj Hospital, Mahidol University, Thailand. This research project is supported by the Siriraj Research Development Fund, Grant number (IO) R016737002, Faculty of Medicine Siriraj Hospital, Mahidol University.

## Declaration of Competing Interest

The authors declare that they have no competing interest with the contents of this article.

## Data Availability

This article contains supporting information in supplementary table and figures. The data will be made available on request. Metabolome data is available as a Mendeley Dataset (DOI: 10.17632/kf7ykbyn25.1). Availability of materials: This study did not generate new unique reagents.
