# Supplementary material for: LC-MS/MS identifies elevated imidazole propionate and gut-derived metabolite alterations in peritoneal dialysis patients
Source: Comput Struct Biotechnol J. 2025 Nov 17;27:5271–80. doi: 10.1016/j.csbj.2025.11.039 (PMC12681520; doi:10.1016/j.csbj.2025.11.039)
Supplement: Supplementary file 1 — Supplementary material [file mmc1.docx]

**Supplementary figures and methods**

**LC-MS/MS Identifies Elevated Imidazole Propionate and Gut-Derived Metabolite Alterations in Peritoneal Dialysis Patients**

Weerawan Manokasemsan^1,2,3,4^, Narumol Jariyasopit^2,3,4^, Kwanjeera Wanichthanarak^2,3^, Patcha Poungsombat^2,4^, Alongkorn Kurilung^2,3^, Suphitcha Limjiasahapong^3,4^, Kajol Thapa^1,3^, Yongyut Sirivatanaukson^2,3,4,5^, Sukit Ruksasuk^2,6^, Thatsaphan Srithongkul^2,6^, Chagriya Kitiyakara^7^, Sakda Khoomrung^1,2,3,4,8,*^

*^1^Department of Biochemistry*, *Faculty of Medicine Siriraj Hospital, Mahidol University, Bangkok, Thailand*

*^2^Siriraj Center of Research Excellent in Metabolomics and Systems Biology (SiCORE-MSB), Faculty of Medicine Siriraj Hospital, Mahidol University, Bangkok, Thailand*

*^3^Siriraj Metabolomics and Phenomics Center, Faculty of Medicine Siriraj Hospital, Mahidol University, Bangkok, Thailand*

*^4^Thailand Metabolomics Association, Bangkok, Thailand*

*^5^Department of Surgery, Faculty of Medicine Siriraj Hospital, Mahidol University, Bangkok, Thailand*

*^6^Division of Nephrology, Department of Medicine, Faculty of Medicine Siriraj Hospital, Mahidol University*

^7^Department of Medicine, Ramathibodi Hospital, Mahidol University, Bangkok, Thailand

*^8^Center of Excellence for Innovation in Chemistry (PERCH-CIC), Faculty of Science, Mahidol University, Bangkok, Thailand*

*Sakda Khoomrung: <https://orcid.org/0000-0001-9461-8597>

Sakda Khoomrung, Email: [sakda.kho@mahidol.edu](mailto:sakda.kho@mahidol.edu)


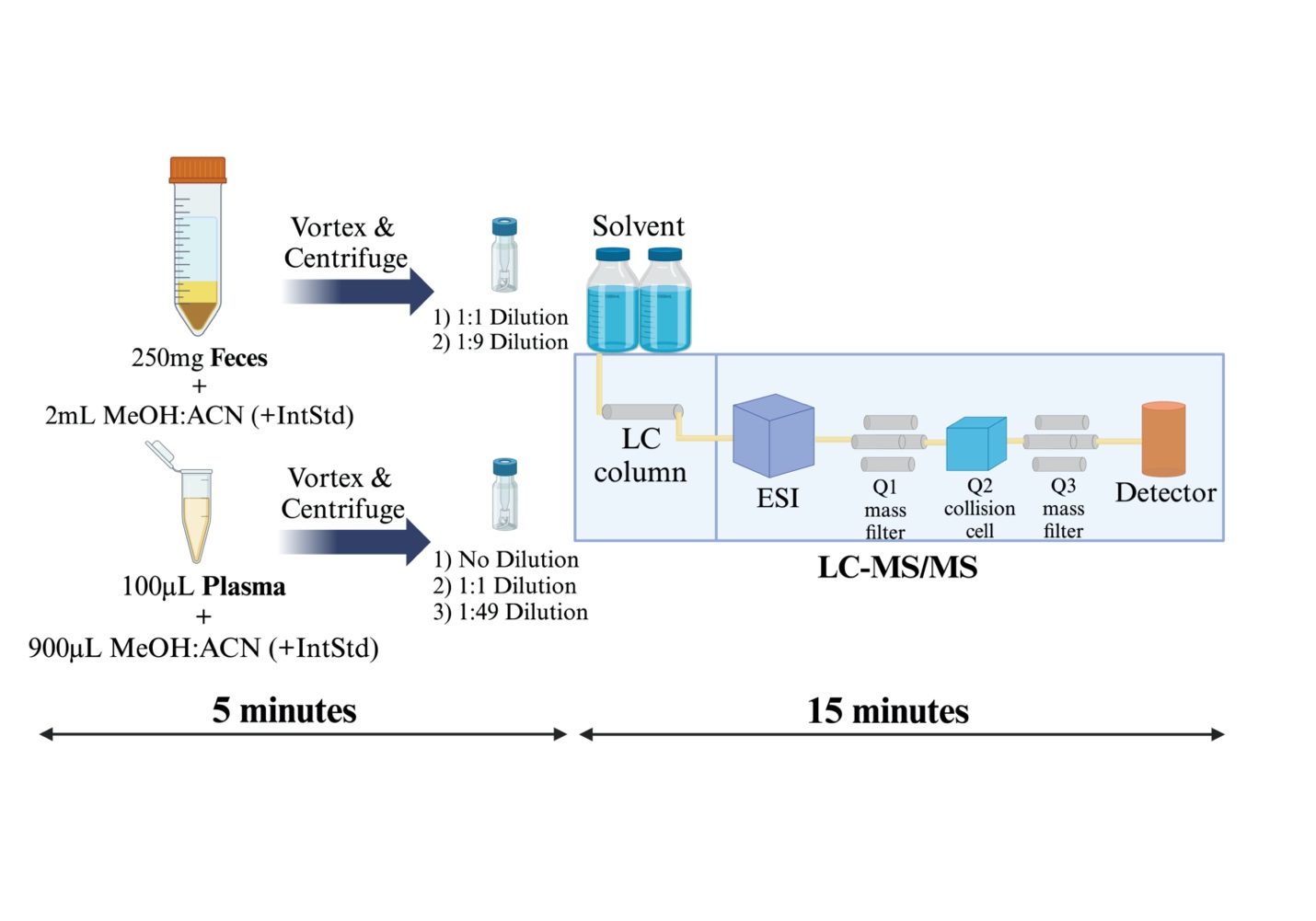


**Figure S1.** Overview of host-microbe co-metabolites analysis with a brief extraction protocol for fecal and plasma samples.


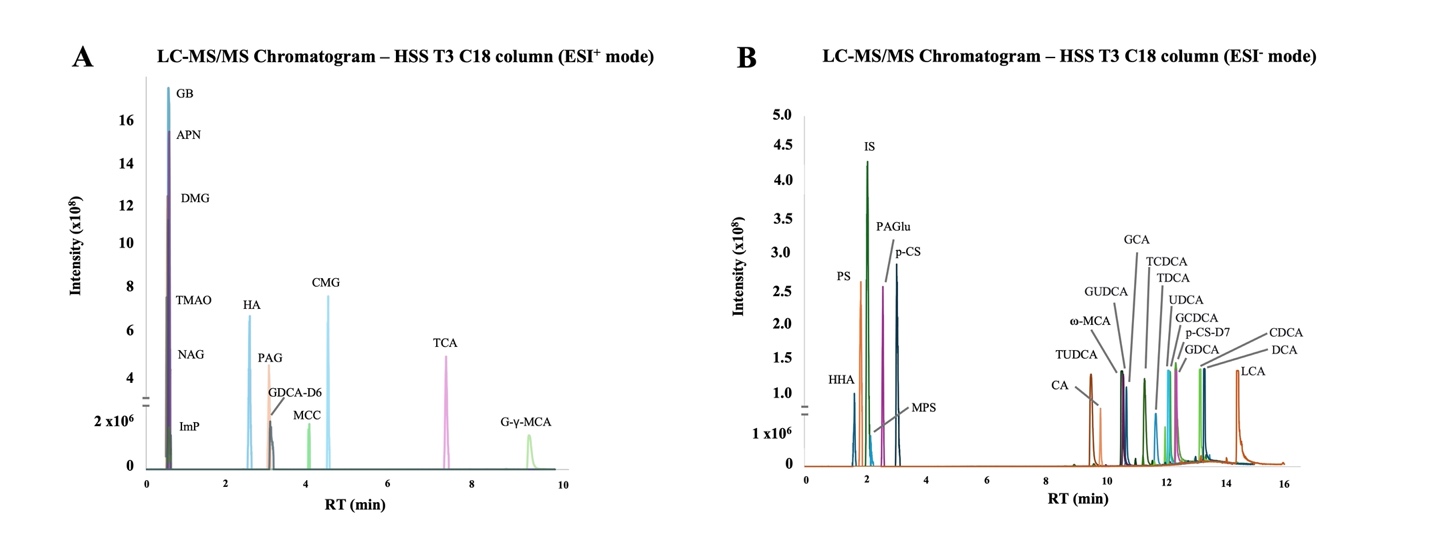


**Figure S2.** Overview of LC-MS/MS chromatogram using HSS T3 C18 reverse phase column. All the single authentic standards are individually analyzed in (A) positive ESI (ESI^+^) and (B) negative ESI (ESI^-^) modes. The method using the HSS T3 C18 column is briefly described in supplementary document S1.


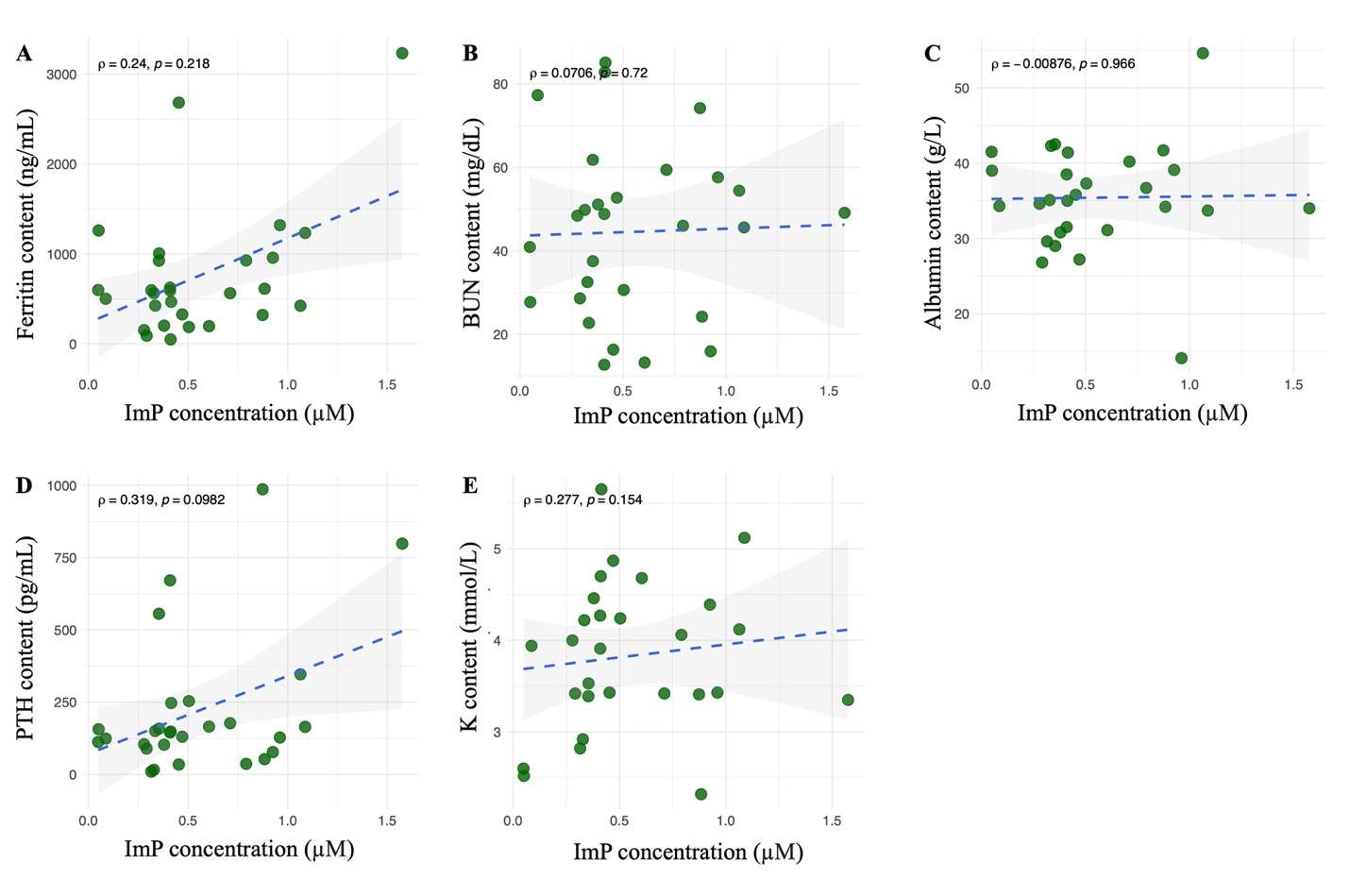


**Figure S3.** The regression plots present the relationship of plasma Imp concentration (μM) and five kidney disease-related clinical parameters: (A) ferritin, (B) BUN, (C) albumin, (D) PTH, and (E) K content. The dashed line signifies the regression line, with the grey area indicating the 95% confidence interval. A significant relationship is denoted by the p-value (p) and Rho (ρ) represents correlation coefficient.

**Supplementary document**

**Supplementary document S1. Identification method using HSS T3 C18 column**

*S1.1 Host-microbe co-metabolites analysis*

To prepare 20 µM of mixed standards solution, 33 mixed UT and BA standards, and 2 internal standards were prepared in methanol. The mixed standard solution was directly injected into LC-MS/MS.

*S1.2 LC-MS/MS analysis*

The analysis was performed using Waters Xevo TQ-S (Waters Corporation, Milford, MA, USA). For the chromatographic conditions, the study followed a previously developed protocol (Garcia-Canaveras et al., 2012). The targeted standards were chromatographically separated using the ACQUITY UPLC T3 C18 column (2.1 mm × 100 mm, 1.7 μm; Waters, Milford, MA, USA). To cover the wide range of analytes, the separation of chromatographic methods was performed using both positive and negative electrospray ionization (ESI). For mobile phase A, 0.1% formic acid in H_2_O. For mobile phase B, 0.1% formic acid in ACN. The injected volume was set at 5 μL, and the column temperature was set at 65°C. The chromatographic gradient was as follows: 0−0.5 min, 95%; 0.5−5.5 min, 95% to 75% A; 5.5−16 min, 75% to 60% A; 16−17.5 min, 60% to 5% A; 17.5−19 min, holds at 5% A; finally, the initial condition was recovered (95% A) and re-equilibrated at 95% A for 2 minutes, with a flow rate of 0.5 mL/min.

The UPLC system was coupled to a TQ-S MS system (Xevo, Waters, Manchester, UK) with an ESI source. In the ESI mode, the following parameters were applied: capillary voltage 2.0 kV; source temperature 120 °C; and desolvation temperature 380 °C. For the gas flow settings, the optimized parameters for the ESI mode were as follows: gas flow rate for desolvation 800 L/Hr; for cone 150 L/Hr; and collision gas was 0.25 mL/min.

**Reference for supplementary document:**

Garcia-Canaveras, J.C., Donato, M.T., Castell, J.V., and Lahoz, A. (2012). Targeted profiling of circulating and hepatic bile acids in human, mouse, and rat using a UPLC-MRM-MS-validated method. J Lipid Res *53*, 2231-2241.
